# Association between bilirubin and biomarkers of metabolic health and oxidative stress in the MARK-AGE cohort

**DOI:** 10.1016/j.isci.2024.110234

**Published:** 2024-06-09

**Authors:** Vanessa Schoissengeier, Lina Maqboul, Daniela Weber, Tilman Grune, Alexander Bürkle, Maria Moreno-Villaneuva, Claudio Franceschi, Miriam Capri, Jürgen Bernhard, Olivier Toussaint, Florence Debacq-Chainiaux, Birgit Weinberger, Efstathios S. Gonos, Ewa Sikora, Martijn Dollé, Eugène Jansen, P. Eline Slagboom, Antti Hervonnen, Mikko Hurme, Nicolle Breusing, Jan Frank, Andrew C. Bulmer, Karl-Heinz Wagner

**Affiliations:** 1 Department of Nutritional Sciences, University of Vienna, Vienna 1090, Austria; 2 Vienna Doctoral School for Pharmaceutical, Nutritional and Sport Sciences (PhaNuSpo), University of Vienna, 1090 Vienna, Austria; 3 Department of Molecular Toxicology, German Institute of Human Nutrition Potsdam-Rehbruecke (DIfE), 14558 Nuthetal, Germany; 4 Food4Future (F4F), c/o Leibniz Institute of Vegetable and Ornamental Crops (IGZ), 14979 Grossbeeren, Germany; 5 German Center for Diabetes Research (DZD), 85764 Neuherberg, Germany; 6 German Diabetes Center (DDZ), Leibniz Center for Diabetes Research, 40225 Düsseldorf, Germany; 7 Molecular Toxicology Group, Department of Biology, University of Konstanz, 78464 Konstanz, Germany; 8 Human Performance Research Centre, Department of Sport Science, University of Konstanz, 78464 Konstanz, Germany; 9 Department of Medical and Surgical Sciences, University of Bologna, 40126 Bologna, Italy; 10 Laboratory of Systems Medicine of Healthy Aging, Institute of Biology and Biomedicine and Institute of Information Technology, Mathematics and Mechanics, Department of Applied Mathematics, Lobachevsky State University, 603005 Nizhny Novgorod, Russia; 11 Interdepartmental Center - Alma Mater Research Institute on Global Challenges and Climate Change - University of Bologna, 40126 Bologna, Italy; 12 BioTeSys GmbH, 73728 Esslingen, Germany; 13 URBC-NARILIS, University of Namur, 5000 Namur, Belgium; 14 Institute for Biomedical Aging Research, Universität Innsbruck, 6020 Innsbruck, Austria; 15 National Hellenic Research Foundation, Institute of Biology, Medicinal Chemistry and Biotechnology, 116 35 Athens, Greece; 16 Laboratory of the Molecular Bases of Ageing, Nencki Institute of Experimental Biology, Polish Academy of Sciences, 00-901 Warsaw, Poland; 17 Centre for Health Protection, National Institute for Public Health and the Environment, 3720BA Bilthoven, the Netherlands; 18 Department of Molecular Epidemiology, Leiden University Medical Centre, 2333 ZC Leiden, the Netherlands; 19 Faculty of Medicine and Health Technology, Tampere University, 33100 Tampere, Finland; 20 Department of Applied Nutritional Science/Dietetics, Institute of Nutritional Medicine, University of Hohenheim, 70599 Stuttgart, Germany; 21 Department of Food Biofunctionality, University of Hohenheim, 70599 Stuttgart, Germany; 22 School of Pharmacy and Medical Sciences, Griffith University, Gold Coast Campus, Brisbane, QLD, Australia; 23 Research Platform Active Ageing, University of Vienna, Vienna 1090, Austria

**Keywords:** pathology, public health, human metabolism

## Abstract

Recent studies have shown that elevated concentrations of unconjugated bilirubin (UCB) may be a protective host factor against the development of noncommunicable diseases (NCDs), whereas low levels of UCB are associated with the opposite effect. The results of this European study, in which 2,489 samples were tested for their UCB concentration using high-performance liquid chromatography (HPLC) and additional data from the MARK-AGE database were used for analysis, provide further evidence that elevated UCB concentrations are linked to a lower risk of developing NCDs and may act as a predictive marker of biological aging as individuals with elevated UCB concentrations showed favorable outcomes in metabolic health and oxidative-stress-related biomarkers. These findings underline the significance of studying individuals with moderate hyperbilirubinemia and investigate UCB routinely, also in the setting of aging, since this condition affects millions of people worldwide but has been underrepresented in clinical research and practice until now.

## Highlights

• Slightly elevated UCB is a protective host factor against the development of NCDs
• Humans with elevated UCB had better metabolic health and oxidative stress markers
• UCB may act as a predictive biomarker of biological aging

## Graphical abstract

**Figure:**
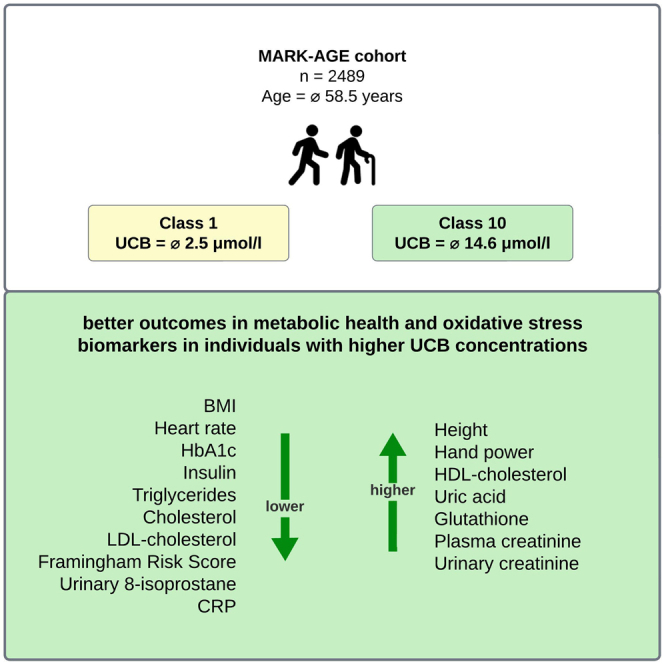

## Introduction

Aging is the most profound risk factor for almost all noncommunicable diseases (NCDs).^1^^,^^2^ Besides this non-modifiable risk factor,^3^ especially metabolic health factors such as high blood pressure, diabetes mellitus, obesity, and high blood cholesterol or oxidative-stress-related parameters also play a key role in developing these diseases.^4^ As NCDs are the leading cause of death worldwide,^3^^,^^5^ it is utterly important to focus on reducing the risk factors associated with these diseases.^5^ Hence, it is important to identify and study protective factors in addition to risk factors, as this can contribute to a healthier society and to an increased quality of life in older adults. Recent studies have shown that elevated concentrations of unconjugated bilirubin (UCB) may be an important protective host factor against the development of NCDs, whereas low levels of UCB are associated with the opposite effect.^6^^,^^7^ Elevated UCB levels are typically found in individuals with Morbus Meulengracht, also known as Gilbert syndrome (GS)^8^ but also in individuals with serum bilirubin in the upper quartiles of the physiological range, which is defined as 5–17 μmol/L.^7^ Originally UCB was thought to be a non-functional, potentially harmful waste product of heme metabolism, as it is neurotoxic at pathological concentrations exceeding the binding capacity of plasma albumin, sometimes found in newborns.^6^ However, there is increasing evidence that UCB is an important modulator of several biological functions in the human body.^6^^,^^9^ In addition to being one of the most potent endogenous antioxidants,^10^^,^^11^^,^^12^ bilirubin is also recognized as a potent immunosuppressant and a selective cell signaling molecule.^12^ In one of the few human studies, Maruhashi et al. showed that individuals with GS had lower levels of malondialdehyde-modified low-density lipoproteins (LDL) and urinary excretion of 8-hydroxy-2′-deoxyguanosine, compared to matched control individuals, as indices of oxidative stress.^13^ We could recently show that GS individuals (*n* = 119) demonstrate a lower BMI; 37% higher antioxidant potential assessed as ferric-reducing ability potential (FRAP); higher advanced oxidation protein products (AOPP); and lower apolipoprotein B, hs-C-reactive protein (CRP), interleukin-6, and interleukin-1β values compared to healthy controls.^14^

A higher total antioxidant status in individuals with GS compared to controls has also been observed in other studies.^15^^,^^16^ In addition, various metabolic health parameters have been associated with UCB. Inverse association between elevated UCB concentration and BMI and triglycerides,^17^ heart rate,^14^ cholesterol, and LDL cholesterol^18^ has been shown, as well as the inverse relationship between elevated total bilirubin concentrations and HbA1c^19^^,^^20^^,^^21^^,^^22^^,^^23^ and insulin.^24^^,^^25^^,^^26^ Therefore, the aim of this secondary analysis of the large MARK-AGE cohort was to investigate whether individuals with elevated UCB concentrations have favorable results in terms of parameters of metabolic health and oxidative stress, compared to individuals with lower UCB concentrations, and whether UCB could serve as a predictor of biological aging.^4^

## Results

### Characteristics of the study population

The characteristics of the study groups are shown in Table 1. The mean age of participants was 58.5 ± 10.6 years, with no significant difference between males and females (*p* = 0.248). Males (*n* = 1128) represented 45.3% of the participants and had higher mean UCB concentrations compared to females. A total of 2,489 participants were divided into the groups as follows: RASIG (*n* = 1179), GO (*n* = 447), and SGO (*n* = 263) (see section study population and sample collection). The RASIG group had the highest mean UCB concentration, GO the second highest, and SGO had the lowest. The mean concentrations of the two liver enzymes alanine transaminase (ALT) and gamma-glutamyl transferase (γ-GT) were within the normal range, indicating that individuals involved did not have severe liver dysfunction.

**Table 1:** Demographic features, metabolic health, and oxidative-stress-related markers of the MARK-AGE study population

Parameters	n	Range	Mean (SD)
UCB (μmol/L)	2489	0.7–36.3	**6.393** (3.61)
Subject Groups			
RASIG	1779	0.7–36.3	**6.571** (3.73)
GO	447	1.7–25.5	**6.083** (3.36)
SGO	263	1.1–18.5	**5.716** (2.99)
Sex			
Female	1361	1.1–36.3	**5.814** (3.20)
Male	1128	0.7–32.4	**7.092** (3.93)
Age (years)	2489	31.6–80.9	**58.49** (10.6)
BMI (kg/m^2^)	2489	15.8–48.9	**26.31** (4.43)
Weight (kg)	2489	42–135	**75.15** (14.8)
Height (cm)	2489	141–200	**168.8** (9.41)
WC (cm)	2489	52–152	**92.62** (12.5)
WHR	2489	0.5–1.27	**0.907** (0.08)
Heart rate (bpm)	2489	38–153	**70.18** (11.1)
BP diastolic (mmHg)	2489	51–137	**81.00** (11.0)
BP systolic (mmHg)	2489	80–270	**136.0** (20.1)
Left hand power (kg)	2478	0–72	**32.79** (10.8)
Right hand power (kg)	2485	0–100	**34.30** (11.1)
HbA1c (%)	2470	4.6–13.1	**6.043** (0.59)
Insulin (μU/mL)	2487	0–65.2	**6.128** (4.77)
Triglycerides (mmol/L)	2326	0.2–12.0	**1.271** (0.87)
Cholesterol (mmol/L)	2327	2.0–11.2	**5.609** (1.03)
HDL cholesterol (mmol/L)	2360	0.5–3.7	**1.537** (0.44)
LDL cholesterol (mmol/L)	2361	0.5–8.3	**3.331** (0.87)
Adiponectin (ng/mL)	2487	1.9–64.5	**14.35** (7.65)
FRS (points)	2246	−1–23	**12.98** (4.17)
ALT (U/l)	2487	5–100	**24.18** (10.3)
γ-GT (U/l)	2346	0–362	**22.36** (28.0)
Ascorbic acid (mg/L)	2489	0.05–62.3	**5.177** (3.65)
Uric acid (mg/L)	2489	13.1–109	**45.45** (12.0)
Glutathione (μmol/L)	2488	435–2090	**1107** (196)
Cysteine (μmol/L)	2488	39.4–291	**143.9** (33.4)
MDA (μmol/L)	2489	0–3.7	**0.322** (0.23)
Protein carbonyls (nmol/mg)	2489	0.3–2.8	**0.582** (0.10)
3-Nitrotyrosine (pmol/mg)	2470	0.1–27	**4.491** (2.80)
Urinary 8-isoprostane (ng/mL)	2480	0.1–40.2	**2.316** (2.32)
Plasma creatinine (μmol/L)	2487	30.5–241	**74.04** (15.9)
Urinary creatinine (pmol/L)	2480	0.6–46.2	**10.35** (5.83)
CRP (mg/L)	2487	0–40.9	**2.199** (3.27)
Arg-Pyr (%)	2441	0.0003–19.2	**0.313** (0.78)
CML (%)	2441	0.0003–9.97	**0.275** (0.80)

Data are presented as mean ± standard deviation. The means are in bold to make them stand out.

UCB, unconjugated bilirubin; RASIG, randomly recruited age-stratified individuals from the general population; GO, GEHA (genetics of healthy aging) offspring; SGO, spouses of GO (GEHA offspring); FRS, Framingham Risk Score; ALT, alanine aminotransferase; γ-GT, gamma-glutamyl transferase; MDA, malondialdehyde; CRP, C-reactive protein; Arg-Pyr, arg-pyrimidine; CML, carboxymethyllysine.

### Differences in biomarkers between Class1 and Class10 and between UCB <10 μmol/L and UCB ≥10 μmol/L

According to the UCB concentrations, participants were divided into 10 equally sized classes (*n* = 248) and the differences between Class1 (UCB range: 0.7–3.0 μmol/L) and Class10 (UCB range: 10.9–36.3 μmol/L) were evaluated. Additionally, UCB subgroups separated by common cutoff points (cutoff: 10 μmol/L and cutoff: 17.1 μmol/L; see Table S1) were analyzed. BMI was significantly lower in both subgroups with higher UCB concentrations. Height was significantly higher in Class10 and in the subgroup with UCB ≥10 μmol/L. Waist-to-hip ratio (WHR) was only significantly higher in the subgroup with UCB ≥10 μmol/L compared to subgroups with lower UCB concentrations. Both groups with higher serum UCB levels had lower heart rates, lower HbA1c, lower insulin, lower triglycerides, lower cholesterol, lower LDL cholesterol, and fewer points on the Framingham Risk Score (FRS). Both groups with higher serum UCB levels had higher left hand power and right hand power. High-density lipoprotein (HDL) cholesterol was significantly higher only in Class10. There were no significant differences in adiponectin and in the liver enzymes ALT and γ-GT. Ascorbic acid and cysteine were only significantly lower in the subgroup with UCB ≥10 μmol/L. Uric acid was only significantly higher in Class10. MDA, protein carbonyls, and 3-nitrotyrosine were non-significantly different in the two UCB subgroups. Both groups with higher serum UCB levels had higher glutathione, plasma creatinine, and urinary creatinine. Both subgroups with higher UCB concentrations demonstrated significantly lower urinary 8-isoprostane and CRP. There were no significant differences in the AGEs measured (Table 2).

**Table 2:** Differences in biomarkers between Class1 and Class10 and between UCB <10 μmol/L and UCB ≥10 μmol/L

Parameters	Total		Class 1		Class 10		*p* value	UCB <10 μmol/L		UCB ≥10 μmol/L		*p* value
	n	Mean (SD)	n	Mean (SD)	n	Mean (SD)		n	Mean (SD)	n	Mean (SD)	
UCB (μmol/L)	2489	**6.393** (3.61)	248	**2.475** (0.43)	248	**14.62** (4.02)	***<0.001***	2181	**5.348** (1.94)	308	**13.79** (3.98)	***<0.001***
Age (years)	2489	**58.49** (10.6)	248	**58.17** (10.5)	248	**58.15** (11.6)	0.703	2181	**58.55** (10.5)	308	**58.05** (11.3)	0.774
BMI (kg/m^2^)	2489	**26.31** (4.43)	248	**27.12** (5.16)	248	**25.67** (4.20)	***0.004***	2181	**26.40** (4.46)	308	**25.69** (4.15)	***0.013***
Weight (kg)	2489	**75.15** (14.8)	248	**75.18** (16.3)	248	**76.10** (15.2)	0.322	2181	**75.05** (14.8)	308	**75.89** (15.0)	0.249
Height (cm)	2489	**168.8** (9.41)	248	**166.3** (8.65)	248	**171.8** (9.68)	***<0.001***	2181	**168.4** (9.31)	308	**171.5** (9.71)	***<0.001***
WC (cm)	2489	**92.62** (12.5)	248	**94.08** (13.6)	248	**92.63** (12.2)	0.349	2181	**92.64** (12.6)	308	**92.51** (12.1)	0.885
WHR	2489	**0.907** (0.08)	248	**0.909** (0.07)	248	**0.918** (0.08)	0.17	2181	**0.905** (0.08)	308	**0.916** (0.08)	***0.013***
Heart rate (bpm)	2489	**70.18** (11.1)	248	**73.05** (10.9)	248	**68.54** (11.3)	***<0.001***	2181	**70.33** (11.1)	308	**69.07** (11.3)	***0.044***
BP diastolic (mmHg)	2489	**81.00** (11.0)	248	**81.65** (10.8)	248	**81.26** (10.4)	0.712	2181	**80.96** (11.0)	308	**81.30** (10.7)	0.597
BP systolic (mmHg)	2489	**136.0** (20.1)	248	**136.7** (21.0)	248	**136.5** (20.4)	0.879	2181	**136.0** (20.1)	308	**135.9** (19.5)	0.849
Left hand power (kg)	2478	**32.79** (10.8)	244	**31.37** (10.8)	247	**36.03** (10.7)	***<0.001***	2171	**32.39** (10.8)	307	**35.63** (10.9)	***<0.001***
Right hand power (kg)	2485	**34.30** (11.1)	248	**32.60** (10.9)	248	**37.44** (10.8)	***<0.001***	2177	**33.90** (11.1)	308	**37.12** (11.0)	***<0.001***
HbA1c (%)	2470	**6.043** (0.59)	243	**6.139** (0.73)	245	**5.918** (0.48)	***<0.001***	2165	**6.060** (0.60)	305	**5.927** (0.50)	***<0.001***
Insulin (μU/mL)	2487	**6.128** (4.77)	247	**7.441** (6.03)	248	**5.033** (2.84)	***<0.001***	2179	**6.274** (4.95)	308	**5.099** (3.01)	***<0.001***
Triglycerides (mmol/L)	2326	**1.271** (0.87)	233	**1.481** (1.41)	228	**1.110** (0.56)	***0.005***	2043	**1.293** (0.90)	283	**1.109** (0.54)	***0.002***
Cholesterol (mmol/L)	2327	**5.609** (1.03)	233	**5.601** (0.96)	228	**5.376** (1.05)	***0.016***	2044	**5.630** (1.02)	283	**5.463** (1.09)	***0.01***
HDL cholesterol (mmol/L)	2360	**1.537** (0.44)	237	**1.441** (0.45)	231	**1.528** (0.44)	***0.012***	2073	**1.534** (0.44)	287	**1.552** (0.44)	0.378
LDL cholesterol (mmol/L)	2361	**3.331** (0.87)	237	**3.393** (0.84)	231	**3.159** (0.89)	***0.001***	2074	**3.348** (0.86)	287	**3.211** (0.93)	***0.003***
Adiponectin (ng/mL)	2487	**14.35** (7.65)	248	**14.54** (8.71)	247	**14.61** (8.00)	0.519	2180	**14.30** (7.62)	307	**14.72** (7.85)	0.269
FRS (points)	2246	**12.98** (4.17)	220	**13.91** (3.63)	211	**11.71** (4.55)	***<0.001***	1979	**13.13** (4.10)	267	**11.80** (4.49)	***<0.001***
ALT (U/l)	2487	**24.18** (10.3)	247	**23.69** (10.4)	248	**23.62** (9.12)	0.721	2179	**24.20** (10.4)	308	**24.06** (9.21)	0.498
γ-GT (U/l)	2346	**22.36** (28.0)	236	**24.41** (33.4)	228	**21.68** (27.0)	0.774	2062	**22.58** (28.4)	284	**20.79** (25.0)	0.754
Ascorbic acid (mg/L)	2489	**5.177** (3.65)	248	**5.610** (5.10)	248	**4.685** (2.91)	0.098	2181	**5.238** (3.73)	308	**4.742** (3.02)	***0.045***
Uric acid (mg/L)	2489	**45.45** (12.0)	248	**43.67** (12.1)	248	**46.75** (12.6)	***0.005***	2181	**45.30** (11.9)	308	**46.50** (12.4)	0.057
Glutathione (μmol/L)	2488	**1107** (196)	248	**1091** (192)	248	**1134** (202)	***0.04***	2180	**1103** (195)	308	**1132** (201)	***0.029***
Cysteine (μmol/L)	2488	**143.9** (33.4)	248	**143.5** (38.5)	248	**138.3** (30.9)	0.258	2180	**144.6** (33.7)	308	**138.8** (30.6)	***0.007***
MDA (μmol/L)	2489	**0.322** (0.23)	248	**0.312** (0.22)	248	**0.322** (0.20)	0.16	2181	**0.321** (0.23)	308	**0.324** (0.19)	0.245
Protein carbonyls (nmol/mg)	2489	**0.582** (0.10)	248	**0.590** (0.17)	248	**0.588** (0.08)	0.191	2181	**0.581** (0.10)	308	**0.590** (0.08)	0.05
3-Nitrotyrosine (pmol/mg)	2470	**4.491** (2.80)	242	**4.577** (3.03)	246	**4.381** (2.39)	0.867	2164	**4.501** (2.84)	306	**4.415** (2.45)	0.704
Urinary 8-isoprostane (ng/mL)	2480	**2.316** (2.32)	246	**2.894** (3.54)	247	**2.055** (1.32)	***<0.001***	2173	**2.354** (2.43)	307	**2.044** (1.31)	***0.001***
Plasma creatinine (μmol/L)	2487	**74.04** (15.9)	247	**70.44** (18.8)	248	**78.46** (15.4)	***<0.001***	2179	**73.48** (15.9)	308	**78.01** (15.3)	***<0.001***
Urinary creatinine (pmol/L)	2480	**10.35** (5.83)	246	**9.645** (5.56)	247	**11.26** (6.07)	***0.002***	2173	**10.21** (5.81)	307	**11.37** (5.86)	***<0.001***
CRP (mg/L)	2487	**2.199** (3.27)	247	**2.664** (3.23)	248	**2.082** (4.04)	***<0.001***	2179	**2.220** (3.12)	308	**2.050** (4.18)	***<0.001***
Arg-Pyr (%)	2441	**0.313** (0.78)	242	**0.260** (0.35)	242	**0.404** (1.29)	0.063	2140	**0.305** (0.71)	301	**0.372** (1.16)	0.092
CML (%)	2441	**0.275** (0.80)	242	**0.191** (0.43)	242	**0.253** (0.87)	0.247	2140	**0.273** (0.78)	301	**0.288** (0.93)	0.373

Data are presented as mean ± standard deviation; *p* values are calculated using Mann-Whitney U test for measuring differences between the subgroups; significant differences are highlighted with bold numbers. Significant p-values are shown in bold and italics.

UCB, unconjugated bilirubin; RASIG, randomly recruited age-stratified individuals from the general population; GO, GEHA offspring; SGO, spouses of GO; FRS, Framingham Risk Score; ALT: alanine aminotransferase; γ-GT, gamma-glutamyl transferase; MDA, malondialdehyde; CRP, C-reactive protein; Arg-Pyr, arg-pyrimidine; CML, carboxymethyllysine.

### Differences in biomarkers between Class1 and Class10, separated by sex

As presented in Table 3, we compared all parameters, separated by sex. Females in Class10 had elevated left hand power and right hand power and also elevated HDL cholesterol compared to females in Class1. Adiponectin, plasma creatinine, and arg-pyrimidine were also significantly higher in females of Class10 compared to Class1. When comparing females within the classes, females in Class10 had lower BMI, weight, waist circumference (WC), BP diastolic, BP systolic, insulin, LDL cholesterol, FRS points, CRP, and carboxymethyllysine (CML).

**Table 3:** Differences in biomarkers between Class1 and Class10, separated by sex

Parameter	Females					Males				
	Class 1		Class 10		*p*-Value	Class 1		Class 10		*p*-Value
	N	Mean (SD)	N	Mean (SD)		N	Mean (SD)	N	Mean (SD)	
UCB (μmol/L)	156	**2.499** (0.39)	91	**14.46** (4.23)	***<0.001***	92	**2.434** (0.49)	157	**14.71** (3.91)	***<0.001***
Age (years)	156	**58.31** (10.6)	91	**56.54** (12.2)	0.329	92	**57.94** (10.4)	157	**59.09** (11.1)	0.263
BMI (kg/m^2^)	156	**26.30** (5.38)	91	**24.40** (4.58)	***0.003***	92	**28.50** (4.48)	157	**26.40** (3.79)	***<0.001***
Weight (kg)	156	**68.57** (14.0)	91	**65.35** (13.2)	***0.042***	92	**86.39** (13.6)	157	**82.34** (12.6)	***0.027***
Height (cm)	156	**161.6** (6.12)	91	**163.5** (6.73)	0.09	92	**174.2** (6.21)	157	**176.6** (7.70)	***0.012***
WC (cm)	156	**90.21** (13.7)	91	**86.53** (12.8)	***0.02***	92	**100.7** (10.7)	157	**96.17** (10.3)	***0.007***
WHR	156	**0.881** (0.07)	91	**0.864** (0.07)	0.09	92	**0.954** (0.05)	157	**0.948** (0.06)	0.486
Heart rate (bpm)	156	**72.97** (9.99)	91	**70.88** (9.56)	0.114	92	**73.20** (12.5)	157	**67.19** (12.0)	***<0.001***
BP diastolic (mmHg)	156	**80.69** (10.0)	91	**77.24** (9.60)	***0.011***	92	**83.29** (11.8)	157	**83.59** (10.2)	0.696
BP systolic (mmHg)	156	**134.5** (21.1)	91	**129.0** (19.1)	***0.026***	92	**140.4** (20.5)	157	**140.9** (19.9)	0.671
Left hand power (kg)	154	**24.86** (5.11)	90	**26.73** (7.18)	***0.031***	90	**42.51** (8.76)	157	**41.36** (8.55)	0.287
Right hand power (kg)	156	**26.40** (5.51)	91	**28.49** (7.77)	***0.033***	92	**43.12** (9.54)	157	**42.62** (8.76)	0.587
HbA1c (%)	154	**6.142** (0.80)	90	**5.984** (0.42)	0.256	89	**6.135** (0.59)	155	**5.880** (0.51)	***<0.001***
Insulin (μU/mL)	156	**6.288** (5.01)	91	**4.516** (2.22)	***0.002***	91	**9.418** (7.06)	157	**5.332** (3.11)	***<0.001***
Triglycerides (mmol/L)	147	**1.211** (1.04)	84	**0.950** (0.39)	0.056	86	**1.941** (1.79)	144	**1.203** (0.63)	***<0.001***
Cholesterol (mmol/L)	147	**5.677** (0.97)	84	**5.577** (1.02)	0.388	86	**5.472** (0.94)	144	**5.259** (1.05)	0.139
HDL cholesterol (mmol/L)	149	**1.595** (0.45)	86	**1.785** (0.45)	***<0.001***	88	**1.181** (0.33)	145	**1.375** (0.35)	***<0.001***
LDL cholesterol (mmol/L)	149	**3.363** (0.85)	86	**3.140** (0.90)	***0.018***	88	**3.444** (0.81)	145	**3.170** (0.88)	***0.014***
Adiponectin (ng/mL)	156	**17.33** (9.39)	91	**19.24** (8.65)	***0.024***	92	**9.811** (4.44)	156	**11.90** (6.18)	***0.008***
FRS (points)	140	**14.74** (3.78)	77	**12.71** (5.50)	***0.02***	80	**12.46** (2.84)	134	**11.13** (3.81)	***0.041***
ALT (U/l)	156	**21.70** (9.30)	91	**20.45** (5.94)	0.574	91	**27.10** (11.3)	157	**25.46** (10.1)	0.193
γ-GT (U/l)	148	**20.57** (36.8)	83	**16.33** (18.6)	0.885	88	**30.87** (25.9)	145	**24.75** (30.4)	***0.005***
Ascorbic acid (mg/L)	156	**5.848** (3.56)	91	**5.235** (3.06)	0.251	92	**5.208** (6.99)	157	**4.367** (2.77)	0.731
Uric acid (mg/L)	156	**38.87** (10.2)	91	**39.22** (10.8)	0.921	92	**51.81** (10.7)	157	**51.11** (11.4)	0.39
Glutathione (μmol/L)	156	**1085** (168)	91	**1119** (200)	0.252	92	**1101** (229)	157	**1142** (203)	0.248
Cysteine (μmol/L)	156	**145.9** (35.9)	91	**143.2** (29.8)	0.601	92	**139.5** (42.5)	157	**135.5** (31.3)	0.955
MDA (μmol/L)	156	**0.295** (0.20)	91	**0.319** (0.21)	0.219	92	**0.340** (0.25)	157	**0.324** (0.19)	0.728
Protein carbonyls (nmol/mg)	156	**0.581** (0.09)	91	**0.589** (0.08)	0.399	92	**0.605** (0.25)	157	**0.587** (0.08)	0.297
3-Nitrotyrosine (pmol/mg)	153	**4.699** (3.34)	90	**4.604** (2.32)	0.368	89	**4.368** (2.41)	156	**4.252** (2.42)	0.74
Urinary 8-isoprostane (ng/mL)	155	**3.119** (3.04)	91	**2.578** (1.84)	0.063	91	**2.512** (4.25)	156	**1.749** (0.74)	0.393
Plasma creatinine (μmol/L)	156	**63.17** (11.7)	91	**67.28** (12.7)	***0.015***	91	**82.91** (22.1)	157	**84.94** (13.0)	0.054
Urinary creatinine (pmol/L)	155	**8.043** (4.39)	91	**9.143** (5.53)	0.289	91	**12.37** (6.26)	156	**12.50** (6.05)	0.854
CRP (mg/L)	156	**2.245** (2.60)	91	**2.046** (3.73)	***0.047***	91	**3.383** (4.00)	157	**2.102** (4.22)	***<0.001***
Arg-Pyr (%)	150	**0.252** (0.42)	89	**0.554** (1.98)	***0.044***	92	**0.272** (0.21)	153	**0.317** (0.58)	0.895
CML (%)	150	**0.209** (0.53)	89	**0.166** (0.40)	0.128	92	**0.162** (0.15)	153	**0.303** (1.05)	0.638

Data are presented as mean ± standard deviation. The means are in bold to make them stand out. Significant p-values are shown in bold and italics.

UCB, unconjugated bilirubin; RASIG, randomly recruited age-stratified individuals from the general population; GO, GEHA (genetics of healthy aging) offspring; SGO, Spouses of GO (GEHA offspring); FRS, Framingham Risk Score; ALT, alanine aminotransferase; γ-GT, gamma-glutamyl transferase; MDA, malondialdehyde; CRP, C-reactive protein; Arg-Pyr, arg-pyrimidine; CML, carboxymethyllysine.

Height, HDL cholesterol, and adiponectin were elevated in males in Class10. Males in Class10 had lower BMI, weight, WC, heart rate, HbA1c, insulin, triglycerides, LDL cholesterol, and FRS points. Males in Class10 had lower γ-GT and CRP compared to Class1. All other parameters did not differ significantly between individuals with higher UCB levels and those with lower UCB levels.

### Differences in biomarkers between Class1 and Class10, in two age subgroups (</≥ 50 years)

All parameters, separated by age with the cutoff 50 years are shown in Table 4. Within the younger subgroup (<50 years, mean age of 42.7 years), Class10 individuals showed in addition to higher UCB levels, elevated height, left hand power, right hand power, HDL cholesterol, and plasma creatinine compared to Class1 individuals. However, at the same time, Class10 individuals had significantly lower BMI, heart rate, BP diastolic, insulin, triglycerides, and FRS points compared to Class1 individuals. Furthermore, γ-GT, protein carbonyls and CRP were also reduced in Class10 individuals. Within the older subgroup (≥50 years, mean age of 63.2 years) Class10 individuals had elevated UCB levels, were significantly older, were taller, had a higher WHR, and had more left hand power and right hand power. Uric acid, glutathione, plasma creatinine, and urinary creatinine were also increased in Class10. Meanwhile, Class10 individuals showed significantly lower heart rate, HbA1c, insulin, FRS points, urinary 8-isoprostane, and CRP compared to Class1 individuals. All other parameters did not differ between the two UCB subgroups.

**Table 4:** Differences in biomarkers between Class1 vs. Class10, in two age subgroups (</≥ 50 years)

Parameter	Age <50 years (*N* = 130)					Age ≥50 years (*N* = 366)				
	Class 1		Class 10		*p*-Value	Class 1		Class 10		*p*-Value
	N	Mean (SD)	N	Mean (SD)		N	Mean (SD)	N	Mean (SD)	
UCB (μmol/L)	58	**2.434** (0.48)	72	**14.91** (4.17)	***<0.001***	190	**2.488** (0.41)	176	**14.50** (3.96)	***<0.001***
Age (years)	58	**43.06** (4.54)	72	**42.70** (4.20)	0.606	190	**62.79** (6.77)	176	**64.48** (6.57)	***0.015***
BMI (kg/m^2^)	58	**25.95** (4.42)	72	**24.11** (3.71)	***0.008***	190	**27.47** (5.33)	176	**26.30** (4.23)	0.088
Weight (kg)	58	**73.17** (16.9)	72	**73.32** (15.8)	0.84	190	**75.79** (16.1)	176	**77.24** (14.9)	0.214
Height (cm)	58	**167.2** (9.15)	72	**173.7** (10.2)	***<0.001***	190	**166.0** (8.50)	176	**171.1** (9.40)	***<0.001***
WC (cm)	58	**89.53** (13.1)	72	**87.18** (11.6)	0.165	190	**95.47** (13.5)	176	**94.86** (11.7)	0.923
WHR	58	**0.886** (0.08)	72	**0.885** (0.07)	0.938	190	**0.915** (0.07)	176	**0.931** (0.07)	***0.039***
Heart rate (bpm)	58	**75.57** (10.6)	72	**67.75** (12.2)	***<0.001***	190	**72.28** (11.0)	176	**68.87** (10.9)	***0.002***
BP diastolic (mmHg)	58	**80.59** (11.5)	72	**76.29** (8.77)	***0.033***	190	**81.98** (10.5)	176	**83.30** (10.3)	0.27
BP systolic (mmHg)	58	**126.3** (16.6)	72	**124.3** (13.9)	0.486	190	**139.8** (21.3)	176	**141.5** (20.6)	0.395
Left hand power (kg)	58	**34.84** (10.5)	72	**38.50** (10.2)	***0.036***	186	**30.28** (10.7)	175	**35.01** (10.8)	***<0.001***
Right hand power (kg)	58	**36.12** (10.6)	72	**40.01** (11.1)	***0.041***	190	**31.53** (10.7)	176	**36.39** (10.6)	***<0.001***
HbA1c (%)	58	**5.979** (0.45)	72	**5.848** (0.41)	0.082	185	**6.189** (0.80)	173	**5.948** (0.50)	***0.002***
Insulin (μU/mL)	58	**7.093** (5.67)	72	**4.696** (2.65)	***<0.001***	189	**7.548** (6.15)	176	**5.171** (2.91)	***<0.001***
Triglycerides (mmol/L)	55	**1.582** (1.67)	67	**0.988** (0.60)	***0.014***	178	**1.449** (1.32)	161	**1.160** (0.54)	0.127
Cholesterol (mmol/L)	55	**5.492** (1.06)	67	**5.216** (0.82)	0.179	178	**5.635** (0.93)	161	**5.443** (1.13)	0.085
HDL cholesterol (mmol/L)	57	**1.373** (0.44)	68	**1.507** (0.34)	***0.014***	180	**1.463** (0.46)	163	**1.536** (0.47)	0.117
LDL cholesterol (mmol/L)	57	**3.344** (0.90)	68	**3.061** (0.77)	0.063	180	**3.409** (0.82)	163	**3.199** (0.93)	***0.008***
Adiponectin (ng/mL)	58	**12.94** (8.52)	72	**13.16** (5.86)	0.155	190	**15.03** (8.73)	175	**15.20** (8.68)	0.787
FRS (points)	53	**10.70** (3.97)	61	**6.660** (4.08)	***<0.001***	167	**14.93** (2.85)	150	**13.77** (2.79)	***<0.001***
ALT (U/l)	58	**23.12** (10.9)	72	**23.06** (9.11)	0.922	189	**23.87** (10.3)	176	**23.85** (9.14)	0.66
γ-GT (U/l)	56	**25.72** (38.2)	65	**15.73** (14.9)	***0.047***	180	**24.00** (31.9)	163	**24.06** (30.2)	0.413
Ascorbic acid (mg/L)	58	**5.354** (3.95)	72	**4.692** (2.86)	0.616	190	**5.689** (5.41)	176	**4.682** (2.94)	0.102
Uric acid (mg/L)	58	**42.27** (13.6)	72	**44.13** (13.6)	0.349	190	**44.10** (11.6)	176	**47.82** (12.0)	***0.002***
Glutathione (μmol/L)	58	**1065** (225)	72	**1104** (176)	0.357	190	**1099** (181)	176	**1146** (210)	***0.044***
Cysteine (μmol/L)	58	**123.1** (33.7)	72	**126.7** (27.1)	0.532	190	**149.7** (37.8)	176	**143.0** (31.1)	0.186
MDA (μmol/L)	58	**0.292** (0.20)	72	**0.287** (0.19)	0.955	190	**0.318** (0.23)	176	**0.336** (0.20)	0.104
Protein carbonyls (nmol/mg)	58	**0.596** (0.30)	72	**0.595** (0.09)	***0.024***	190	**0.588** (0.10)	176	**0.585** (0.08)	0.8
3-Nitrotyrosine (pmol/mg)	57	**4.493** (2.45)	72	**4.532** (2.67)	0.957	185	**4.603** (3.19)	174	**4.319** (2.26)	0.876
Urinary 8-isoprostane (ng/mL)	58	**2.064** (0.85)	72	**2.042** (1.02)	0.379	188	**3.151** (3.98)	175	**2.060** (1.43)	***<0.001***
Plasma creatinine (μmol/L)	58	**66.67** (13.8)	72	**78.39** (14.2)	***<0.001***	189	**71.60** (20.0)	176	**78.49** (16.0)	***<0.001***
Urinary creatinine (pmol/L)	58	**10.43** (5.94)	72	**11.86** (6.50)	0.179	188	**9.402** (5.43)	175	**11.02** (5.89)	***0.008***
CRP (mg/L)	58	**1.996** (2.27)	72	**1.827** (4.36)	***0.004***	189	**2.870** (3.45)	176	**2.186** (3.91)	***0.015***
Arg-Pyr (%)	58	**0.280** (0.62)	71	**0.288** (0.39)	0.215	184	**0.253** (0.21)	171	**0.452** (1.51)	0.135
CML (%)	58	**0.155** (0.14)	71	**0.188** (0.46)	0.153	184	**0.203** (0.49)	171	**0.280** (0.99)	0.754

Data are presented as mean ± standard deviation.

UCB, unconjugated bilirubin; RASIG, randomly recruited age-stratified individuals from the general population; GO, GEHA (genetics of healthy aging) offspring; SGO, spouses of GO (GEHA offspring); FRS, Framingham Risk Score; ALT, alanine aminotransferase; γ-GT, gamma-glutamyl transferase; MDA, malondialdehyde; CRP, C-reactive protein; Arg-Pyr, arg-pyrimidine; CML, carboxymethyllysine.

### Correlations with UCB concentrations

As shown in Table 5, UCB concentrations showed positive significant correlations with height, WHR, left hand power, right hand power, glutathione, plasma creatinine, urinary creatinine, uric acid, and arg-pyrimidine.

**Table 5:** Correlations with UCB concentrations

Parameters	n	r	*p* Value
Age (years)	2489	−0.027	0.177
BMI (kg/m^2^)	2489	−0.095	***<0.001***
Weight (kg)	2489	0.02	0.33
Height (cm)	2489	0.172	***<0.001***
WC (cm)	2489	−0.035	0.085
WHR	2489	0.04	***0.048***
Heart rate (bpm)	2489	−0.088	***<0.001***
BP diastolic (mmHg)	2489	0.01	0.608
BP systolic (mmHg)	2489	−0.01	0.611
Left hand power (kg)	2478	0.15	***<0.001***
Right hand power (kg)	2485	0.146	***<0.001***
HbA1c (%)	2470	−0.102	***<0.001***
Insulin (μU/mL)	2487	−0.114	***<0.001***
Triglycerides (mmol/L)	2326	−0.104	***<0.001***
Cholesterol (mmol/L)	2327	−0.088	***<0.001***
HDL cholesterol (mmol/L)	2360	0.02	0.334
LDL cholesterol (mmol/L)	2361	−0.083	***<0.001***
Adiponectin (ng/mL)	2487	−0.001	0.952
FRS (points)	2246	−0.157	***<0.001***
ALT (U/l)	2487	0.013	0.521
γ-GT (U/L)	2346	0.003	0.899
Ascorbic acid (mg/L)	2489	−0.063	***0.002***
Uric acid (mg/L)	2489	0.06	***0.003***
Glutathione (μmol/L)	2488	0.068	***<0.001***
Cysteine (μmol/L)	2488	−0.066	***<0.001***
MDA (μmol/L)	2489	0.003	0.866
Protein carbonyls (nmol/mg)	2489	0.039	0.052
3-Nitrotyrosine (pmol/mg)	2470	−0.017	0.4
Urinary 8-isoprostane (ng/mL)	2480	−0.071	***<0.001***
Plasma creatinine (μmol/L)	2487	0.142	***<0.001***
Urinary creatinine (pmol/L)	2480	0.092	***<0.001***
CRP (mg/L)	2487	−0.069	***<0.001***
Arg-Pyr (%)	2441	0.04	***0.047***
CML (%)	2441	0.022	0.269

Data are presented as mean ± standard deviation.

UCB, unconjugated bilirubin; RASIG, randomly recruited age-stratified individuals from the general population; GO, GEHA (genetics of healthy aging) offspring; SGO, spouses of GO (GEHA offspring); FRS, Framingham Risk Score; ALT, alanine aminotransferase; γ-GT, gamma-glutamyl transferase; MDA, malondialdehyde; CRP, C-reactive protein; Arg-Pyr, arg-pyrimidine; CML, carboxymethyllysine.

UCB concentrations showed negative significant correlations with BMI, heart rate, HbA1c, insulin, triglycerides, cholesterol, LDL cholesterol, FRS points, cysteine, ascorbic acid, urinary 8-isoprostane, and CRP.

## Discussion

The aim of this secondary analysis of data from the MARK-AGE study was to investigate whether individuals with elevated unconjugated bilirubin concentrations had beneficial biomarkers in terms of metabolic health parameters and oxidative-stress-related biomarkers. As these parameters have been linked to several age-related diseases,^4^ it raises the question whether UCB could be considered a valid biomarker of healthy aging. A total of 2,489 individuals were included in this analysis, 45.3% males and 54.7% females. Class10 (*n* = 248) consisted of 63.3% males and 36.7% females, reflecting the higher prevalence of GS in males.^27^ Furthermore, the fact that serum bilirubin levels are physiologically higher in males than in females^7^ was reflected in our results, as males had a mean UCB concentration of 7.1 μmol/L and females of 5.8 μmol/L (Tables S2 and S3). When broken down into the two classes we have used for the evaluation, similar UCB levels were found when separated by sex (Tables 3 and S3). This might be due to the smaller subgroup sizes.

### UCB and metabolic health

We showed that in individuals with higher UCB concentration (Class10) BMI, heart rate, HbA1c, insulin, triglycerides, cholesterol, LDL cholesterol, and FRS were significantly lower and that height, left and right hand power, and HDL cholesterol were significantly higher, compared to individuals with lower UCB concentrations (Class1). Most of these associations are also shown when separated by sex (Table 3) and within the two age subgroups (Table 4).

### Association between UCB and age

The mean age of the individuals was 58.5 years and did not differ significantly between Class1 and Class10 or females and males, which is important for the interpretation of the results, since we also looked at sex and age differences. As shown in Tables S4 and S5, UCB levels were significantly lower in the subgroup ≥50 years (6.8 μmol/L vs. 6.3 μmol/L, *p = 0.015*) and within Class10, UCB concentrations also had a tendency to decrease with age (14.9 μmol/L vs. 14.5 μmol/L).

### Association between UCB and anthropometric parameters

Height was positively correlated with UCB concentrations. This difference was also significant within males (Table 3) and when comparing the two age subgroups (Table 4). The correlation between weight and UCB was not significant. However, the trend of both measurements reflects the inverse correlation between UCB and BMI (Table 5). WC showed a tendency to decrease with elevated UCB concentrations (Class10) but this finding was not significant. Supportive of our findings, Seyed Khoei et al. showed that individuals in the highest tercile of UCB were taller and slimmer, compared to the lowest tercile of UCB. They showed lower weight, BMI, WC, and fat mass.^17^

### Association between UCB and cardiovascular parameters

Diastolic and systolic BP showed a tendency to decrease with elevated UCB concentrations (Class10), but these findings were not significant. However, the correlation between heart rate and UCB was significantly negative (Table 5). Not only is increased heart rate associated with elevated blood pressure but also with increased risk of hypertension.^28^ Consistent findings by Wang et al. showed that serum bilirubin was inversely associated with systolic BP and the prevalence of hypertension by inactivating and inhibiting the synthesis of reactive oxygen species in vascular cells. Therefore, strategies aimed at increasing the bioavailability of circulating and tissue bilirubin or mimicking bilirubin’s antioxidant properties may have a significant impact on prevention and control of hypertension as well as coronary heart disease.^29^

### Association between UCB and grip strength

Hand power in individuals with higher UCB concentrations was increased, which was highly significant within the two UCB subgroups and within females (Tables 2 and 3). The association was even greater in the older subgroup (Table 4). Grip strength and its potential as a biomarker has been widely discussed, and Forrest et al. recommended grip strength as a useful indicator for overall health.^30^ A recent review concluded that there is sufficient evidence to support the use of grip strength as an explanatory or predictive biomarker of specific outcomes, including generalized strength and function, bone mineral density, fractures and falls, disease status and comorbidity load, hospital-related variables, or mortality.^31^

### Association between UCB and lipid metabolism parameter

HbA1c, insulin, triglycerides, cholesterol, and LDL cholesterol were negatively correlated with UCB concentrations. The correlation between HDL cholesterol and UCB was not significant, but it was positively associated with elevated UCB levels. These results have already been investigated providing supportive results, such as inverse association between elevated UCB concentration and BMI and triglycerides,^17^ heart rate,^14^ and total and LDL cholesterol.^18^ It has also been reported by other investigators that there is an inverse relationship between elevated total bilirubin concentrations and HbA1c^19^^,^^20^^,^^21^^,^^22^^,^^23^ and insulin.^24^^,^^25^^,^^26^ In line with our findings, other studies have previously shown inverse relationships between bilirubin concentrations and metabolic health parameters. In a clinical study by Takei et al., univariate analysis showed that a lower bilirubin concentration was significantly correlated with higher BMI, WC, triglycerides, uric acid, creatinine, visceral fat area, and lower HDL cholesterol. Their data also showed that biliverdin administration alleviated insulin resistance by ameliorating inflammation and the dysregulation of adipocytokine expression in adipose tissues of DIO mice, and therefore, they concluded that bilirubin may protect against insulin resistance by ameliorating visceral obesity and adipose tissue inflammation.^24^ Liu et al. provide an explanation of the underlying mechanism linking insulin and bilirubin. They found that short-term bilirubin treatment was associated with a decrease in total cholesterol and an increase in PPARγ and adipokines in DIO mice. These results provide mechanistic evidence that bilirubin or altered bilirubin metabolism (e.g., partial UGT1A1 inhibitors) may be useful as a therapeutic approach to reduce obesity and improve insulin resistance and glucose tolerance.^25^ Hana et al. recently provided evidence as to why GS individuals are leaner and protected against chronic metabolic diseases. To date, protection against cardiovascular disease (CVD) and type 2 diabetes (T2D) in GS individuals has been primarily linked to their beneficial lipid profile (low TG). However, in this study we showed that individuals with mild hyperbilirubinemia have increased lipid catabolism, which is partly responsible for the advantageous lipid phenotype of GS. In addition, there we demonstrated increased lipid metabolism in GS subjects, which was supported by increased PPARα, AMPK, and TH levels and decreased insulin levels, concluding that enhanced lipid metabolism in GS appears to be the key strategy for the protective role of bilirubin against obesity, dyslipidemia, type 2 diabetes mellitus (DMT2), and CVD. As a result, bilirubin was highlighted as a promising future target in obese and dyslipidemic patients.^32^

Recent studies have also shown that bilirubin has a hormonal function by binding to the peroxisome-proliferator-activated receptor-α (PPARα), a nuclear receptor that drives the transcription of genes to control adiposity.^12^^,^^33^^,^^34^ UCB binds directly to PPARα, and this interaction occurs at physiological bilirubin concentrations (10–25 μmol/L) but also at pathological levels (>100 μmol/L).^12^ Stec et al. showed in a very recent study that 50 μmol/L bilirubin substantially decreased lipid accumulation in 3T3-L1 cells and also enhanced PPARα activity. These results support that activation of PPARα in adipocytes increases genes involved in fatty acid oxidation and decreases *de novo* lipogenic enzymes, which shows the importance of understanding these processes in obesity management.^34^

As part of the BiliHealth study, Mölzer et al. aimed to explain the compelling differences concerning body composition and overall metabolic health between GS individuals and controls. Through a case-control study (*n* = 120), it was demonstrated that GS individuals had significantly higher rates of phospho-AMPK α1/α2, -Ppar α/γ, and of PgC 1α. AMPK α1 gene expression was equal between the groups, indicating a boosted AMPK pathway in response to fasting in GS individuals. An improved health status in GS individuals was confirmed, as they had significantly lower BMI, glucose, insulin, C-peptide, and triglyceride levels. This provides evidence that the energy and macronutrient metabolic response to fasting are clearly boosted in GS. This may explain why individuals with GS are leaner and metabolically healthier and thus less likely to contract metabolic diseases or die prematurely thereof.^35^ Dullaart et al. investigated the relationship between bilirubin and adiponectin and whether the association between incident CVD and bilirubin is modified by adiponectin. They concluded that bilirubin is positively related to adiponectin, but the association of bilirubin with CVD risk is unaffected by adiponectin.^36^ Adiponectin is an anti-inflammatory adipokine with beneficial effects in a variety of CVDs. And a more recent study also found a significant positive association between total bilirubin and adiponectin. This holds significance since both bilirubin and adiponectin are negatively correlated with obesity and BMI.^37^

### Association between UCB and the risk for cardiovascular diseases

Considering all the abovementioned parameters and how they relate to UCB, it is likely that individuals with higher UCB concentrations have better metabolic health status. This leads to the assumption that these individuals also have a lower risk of many chronic diseases, as these parameters contribute to their development. This assumption was supported by the calculated Framingham 10-year risk score. We observed a decreased risk of cardiovascular events in both subgroups with higher UCB concentrations, compared with Class1 or the subgroup with UCB levels <10 μmol/L (Table 2). The observed difference can also be seen when comparing females and males (Table 3) and when comparing the two age subgroups (Table 4). Increased total bilirubin concentrations are associated with a decrease in FRS, as also shown by Kim et al. in a Korean population^38^ and by Leem et al. in asymptomatic patients with type 2 diabetes.^39^ In the context of CVD, the antioxidant potential of bilirubin is important, as bilirubin inhibits the oxidation of low-density lipoprotein (LDL). This represents one hypothesis in CVD pathogenesis.^6^ Another study also showed that individuals with GS had a reduced prevalence of ischemic heart disease compared with the general population (2% vs. 12.1%). They concluded that chronic hyperbilirubinemia may prevent the development of ischemic heart disease by increasing serum antioxidant capacity.^16^

### UCB and oxidative stress

We could show that in individuals with higher UCB concentration (Class10), uric acid, glutathione, plasma creatinine, and urinary creatinine were significantly higher, and on the other hand, urinary 8-isoprostane and CRP were significantly lower, compared to individuals with lower UCB concentrations (Class1). The correlation between UCB and these parameters was similar, but showed additionally negative correlations between UCB and ascorbic acid and cysteine (Table 5). These differences were also found in the older subgroup (Table 4), but not in the younger subgroup and when separated by sex (Table 3).

### Association between UCB and antioxidants

In addition to endogenous antioxidants such as glutathione, cysteine, and uric acid, exogenous antioxidants are also required to counteract oxidative stress. For example, ascorbic acid is one of the most powerful antioxidants of dietary origin. It is known that a high intake of fruits and vegetables is associated with a high plasma concentration of ascorbic acid.^4^^,^^40^ Therefore, high levels of antioxidants, such as ascorbic acid or uric acid, which also reflect nutritional patterns, are not automatically the result of compensated stress.^41^ Here, we showed that ascorbic acid and cysteine were negatively correlated, whereas uric acid and glutathione were positively correlated with UCB. In individuals with higher UCB concentrations (Class10), uric acid and glutathione were significantly higher, compared to Class1. Similar to these findings, Boon et al. showed significantly higher reduced glutathione levels and non-significantly lower uric acid levels in individuals with GS.^10^ Another study also showed that uric acid was inversely associated with serum bilirubin concentration.^24^ However, so far there are no data investigating potential effects of elevated UCB levels on measures of ascorbic acid or cysteine.

### UCB and oxidation products

There were no significant differences between MDA and UCB classes, sex, and age. By contrast, Movahed et al. found a strong inverse relationship between serum bilirubin concentrations and MDA, which is a highly reactive metabolite of free-radical-induced lipid peroxidation.^42^ Wagner et al. investigated MDA, besides other oxidation products, such as oxLDL, AOPP, and FRAP, in GS individuals compared to healthy controls. GS individuals (*n* = 119) demonstrated a lower BMI and a lower resting heart rate, 37% higher antioxidant potential assessed as FRAP, and higher AOPP compared to healthy controls. The findings contribute to the explanation of why GS serves as an important protector in the pathogenesis of metabolic, oxidative-stress-related diseases.^14^ No significant differences were found regarding UCB and protein carbonyl and 3-nitrotyrosine, but both had a tendency to be lower in individuals with higher UCB levels. Boon et al. also found significantly lower protein carbonyl concentrations in individuals with GS compared to controls.^10^ In our study, urinary 8-isoprostane levels were significantly lower in individuals with higher UCB, but no comparative data were found regarding the correlation between UCB and urinary 8-isoprostane. Individuals with higher UCB concentrations (Class10) showed significantly higher plasma and urinary creatinine compared to those with lower UCB concentrations. In line with our results, Santhanam et al. found a significant positive correlation between serum total bilirubin and creatinine in an analysis of the cross-sectional data from NHANES.^43^ Contrarily, Takei et al. conducted a clinical study and showed that lower bilirubin concentration was significantly correlated with higher creatinine.^24^ As there is no further literature on this relationship, it is not known whether this elevation is a protective mechanism or simply a marker of a widespread metabolic abnormality.^43^

### UCB and CRP

High-sensitivity C-reactive protein (hs-CRP) is a marker of systemic inflammation and a predictor of type 2 diabetes and CVDs.^44^ Furthermore, increased CRP values are associated with various diseases, particularly CVD, DMT2, and metabolic syndrome.^45^ In our study cohort, the mean CRP concentration was 2.2 (±3.27) mg/L, which is within the reference range (normal <3 mg/L).^46^ Our analysis revealed a significant inverse correlation between UCB and CRP. The inverse relationship was also significant when comparing Class1 and Class10 (Table 2), within females and males (Table 3) and within the two age subgroups (Table 4). Ohnaka et al. showed an inverse association of serum bilirubin levels with hs-CRP in a large cross-sectional study of middle aged and elderly Japanese men and women.^21^ A more recent study also showed that serum bilirubin levels were negatively associated with serum hs-CRP levels.^47^

### UCB and AGEs

Arg-pyr and carboxymethyllysine (CML) were both positively associated with UCB, but only arg-pyr (%) showed a significant positive correlation with UCB (Table 5). No comparative literature could be found on the link between arg-pyr and unconjugated bilirubin, except for Kalousová et al., who showed that serum levels of CML were significantly lower in people with GS than in normobilirubinemic controls.^48^ However, AGEs in general have been linked to diabetes and obesity, as well as other diseases, as they increase with aging.^49^

### Conclusion

We conclude that in this secondary analysis of data from the MARK-AGE cohort, individuals with elevated UCB concentrations were strongly associated with beneficial outcomes in metabolic health and oxidative-stress-related parameters, compared to individuals with lower UCB concentrations. We have provided further evidence that elevated UCB levels are a protective factor against the development of NCDs, as individuals with elevated UCB levels are better protected against cell and DNA damage, which in turn affects the aging process and life expectancy. Therefore, we are confident that UCB could be used as a predictive marker of aging.

### Limitations of the study

The MARK-AGE study was a very large European study focusing on biomarkers of aging, thus a strength of this analysis is the large number of individuals, which allowed the study population to be divided into subgroups according to the UCB concentration, with a high number of individuals in every subgroup. Another strength was the sex and age stratification. In addition, bilirubin was measured as UCB using high-performance liquid chromatography (HPLC) and not as total bilirubin with a diazo method, as usually performed in the clinical setting.^14^ A general limitation of observational datasets and secondary analyses is the amount of missing data for some variables and the fact that additional parameters cannot be collected retrospectively. However, the number of missing values was very low for the analysis presented here. For body composition only BMI was reported and no other parameters, such as fat mass.

## STAR★Methods

### Key resources table

**Table:** 

REAGENT or RESOURCE	SOURCE	IDENTIFIER
**Biological samples**		

Serum samples of the MARK-AGE study cohort	MARK-AGE Biobank at University of Hohenheim^50^^,^^51^	N/A

**Chemicals, peptides, and recombinant proteins**		

Glacial acetic acid	Sigma-Aldrich	Cat#PHR1748
Dimethyl sulfoxide	Sigma-Aldrich	Cat#34869
Dioctylamine	Sigma-Aldrich	Cat#D201146
HPLC grade methanol	Sigma-Aldrich	Cat#34860
ROTISOLV HPLC Gradient Grade	Carl Roth	Cat#A511.2
Bilirubin	Sigma-Aldrich	Cat#B4126

**Software and algorithms**		

IBM SPSS Statistics, Version 28.0	IBM Statistics	N/A

**Other**		

Nexera HPLC/UHPLC Setup	Shimadzu	N/A
C18 HPLC-column	Fortis	N/A

### Resource availability

#### Lead contact

Further information and requests for resources and reagents should be directed to and will be fulfilled by the lead contact, Karl-Heinz Wagner (karl-heinz.wagner@univie.ac.at).

#### Materials availability

This study did not generate new unique reagents.

#### Data and code availability

• All data reported in this paper will be shared by the lead contact upon request.
• This paper does not report original code.
• Any additional information required to reanalyze the data reported in this paper is available from the lead contact upon request.

### Experimental model and study participant details

#### Study population and sample collection

The MARK-AGE study was conducted in accordance with the Declaration of Helsinki and with the informed written consent of each participant (for details see Bürkle et al.^52^ and Weber et al.^53^). The study population consisted of about 3,200 individuals representing different geographical regions of Europe, with recruitment centers in Germany, Belgium, Poland, Greece, Austria, Italy and Finland.^52^ The study population covered the age range of 31.6–80.9 years with a mean age of 58.5 ± 10.6 years, with no significant difference between males and females. The characteristics of the study population are shown in more detail in Table 1. These individuals were then divided into three large subject groups.The first group consisted of 2,262 randomly recruited age-stratified individuals from the general population (RASIG) with an equal number of men and women and similar number of individuals in each age classification. The second group (*n* = 528) were individuals born from a long-living parent belonging to a family with long-living sibling(s). As one parent had already been recruited to the Project GEHA (genetics of healthy aging), they were referred to as GEHA offspring (GO). As GO are predicted to age at a slower rate than the average population, they were compared with their spouses as lifestyle controls. This third group was designated Spouses of GEHA offspring (SGO) and consisted of 305 individuals.^52^ Recruitment procedures and the collection of anthropometric, clinical, demographic data and behavioral data have already been reported.^4^^,^^52^^,^^54^^,^^55^ Details of the analytical methods for the determination of several markers (glutathione, cysteine, ascorbic acid, uric acid, malondialdehyde, protein carbonyls, 3-nitrotyrosine) have been described by Weber et al.^4^ Anticoagulated whole blood was obtained by phlebotomy after an overnight fast. Samples of plasma, peripheral blood mononuclear cells and whole blood from different recruitment centers were shipped on dry ice to the MARK-AGE Biobank at the University of Hohenheim, Stuttgart, Germany.^50^^,^^51^ For this secondary analysis, a total of 2673 coded serum samples were sent to the Department of Nutritional Sciences, University of Vienna, Austria on dry ice and stored at −80° until used.^50^ Concentrations of unconjugated bilirubin were measured using HPLC from February to August 2022.

### Method details

#### Determination of unconjugated bilirubin

Unconjugated bilirubin was determined in serum samples following a well-established protocol.^18^^,^^56^^,^^57^ A high-performance liquid chromatography (Shimadzu Nexera HPLC/UHPLC, Vienna, Austria), equipped with a spectrophotometric detector (Shimadzu Nexera HPLC/UHPLC - SPD-40V UV-VIS) and a Fortis C18 HPLC-column (4.6 × 150 mm, 3 μm) with a Phenomenex SecurityGuard cartridge for C18 HPLC columns (4 × 3 mm) was used^18^; modified from Brower at al..^58^ The column was perfused with an isocratic mobile phase containing glacial acetic acid (6.01 g/L) and 0.1 M n-dioctylamine in HPLC grade methanol/water (96.5/3.5%).^59^ UCB was extracted from samples by mixing 50 μL serum with 200 μL mobile phase. After centrifugation (4°C, 14000 rpm, 10 min), 120 μL of the supernatant was injected to the HPLC at a flow rate of 1 mL/min. Retention time (rt) of the IXa peak was 8–9 min. Bilirubin (purity ≥98%, Sigma Aldrich) was used as an external standard (3.3% IIIα, 92.8% IXα, and 3.9% XIIIα isomers, 450 nm)^59^ and a dilution series was prepared and measured. R^2^ for all external standard samples across all runs (*n* = 63), was 0.999 ± 0.006, with a coefficient of variation (CV) of 0.57%, and the CV of the retention time was 1.95%. Additionally, two quality control (QC) samples of human serum of known concentration were evaluated per analysis as internal standards. The QCs were used as reference plasma samples to measure the reliability, repeatability, and accuracy of the data.

### Quantification and statistical analysis

#### Biomarkers used in the statistical analysis

UCB concentrations were matched to the MARK-AGE dataset using the SampleID and searched for duplicate cases. Out of 2673 samples, 162 samples were excluded due to missing values (such as age, sex, weight), 12 samples were excluded due to implausible values. To exclude participants with liver diseases, 8 samples were excluded because of exclusionary and missing information regarding problems with the liver and 2 more samples had to be excluded due to high levels of common liver parameters (γ-GT and ALT). Statistical analysis was performed with 2489 samples and markers referring to different fields. Anthropometric characteristics were described by assessing BMI, weight, height, waist circumference (WC), waist to hip ratio (WHR), heart rate, and diastolic and systolic blood pressure (BP). For grip strength we used right and left hand power. Available variables for glucose and lipid metabolism were HbA1c, insulin, triglycerides, cholesterol, HDL cholesterol, LDL cholesterol, adiponectin. In addition, the Framingham risk score (FRS)^60^ was calculated for all data to provide a better insight into each individual’s long-term risk of cardiovascular disease. To assess individual status of oxidative stress, ascorbic acid, uric acid, glutathione, cysteine, malondialdehyde (MDA), protein carbonyls, 3-nitrotyrosine, plasma and urinary creatinine and C-reactive protein (CRP) were measured. For the assessment of advanced glycation endproducts (AGEs), we measured arg-pyrimidine (Arg-Pyr) and carboxymethyllysine (CML).

#### Statistical analysis

All statistical analyses were performed using SPSS Statistics (IBM Statistics, version 28). Prior to analysis, missing data had been excluded (as described in the section biomarkers used in the statistical analysis). Statistically significant differences were considered at *p < 0.05.* The Kolmogorov-Smirnov test was used to determine normal distribution. For comparison of two groups, the Mann–Whitney U test (non-parametric data) was applied. Correlations between UCB concentrations and other variables were analyzed by Pearson correlation. We decided *a priori* to run all models separately for males and females, as serum bilirubin levels are physiologically higher in males than in females.^7^ As age-related effects associated with mild hyperbilirubinemia on indicators of metabolic health have been observed in previous studies,^61^^,^^62^ we also tested the age cut-off of 50 years. Participants were divided into 10 equally sized classes (*n* = 248) according to the UCB concentrations. In addition, we also analyzed UCB subgroups, separated by common cut-off points, once with a cut-off of 10 μmol/L and once with a cut-off of 17.1 μmol/L (usually seen in people with GS). Demographic characteristics were described using means ± standard deviations (SD) for continuous variables.
