# Supplementary material for: Association between bilirubin and biomarkers of metabolic health and oxidative stress in the MARK-AGE cohort
Source: iScience. 2024 Jun 9;27(7):110234. doi: 10.1016/j.isci.2024.110234 (PMC11253506; doi:10.1016/j.isci.2024.110234)
Supplement: Document S1. Tables S1–S5 [file mmc1.pdf]

iScience, Volume *27*

Supplemental information

Association between bilirubin and biomarkers of metabolic health and oxidative stress in the MARK-AGE cohort

Vanessa Schoissengeier, Lina Maqboul, Daniela Weber, Tilman Grune, Alexander Bürkle, Maria Moreno-Villaneuva, Claudio Franceschi, Miriam Capri, Jürgen Bernhard, Olivier Toussaint, Florence Debacq-Chainiaux, Birgit Weinberger, Efstathios S. Gonos, Ewa Sikora, Martijn Dollé, Eugène Jansen, P. Eline Slagboom, Antti Hervonnen, Mikko Hurme, Nicolle Breusing, Jan Frank, Andrew C. Bulmer, and Karl-Heinz Wagner

Supplemental Information

Table S1: *Differences in biomarkers between UCB < 17.1 µmol/l and UCB ≥ 17.1 µmol/l, related to Table 2*

Parameters	Total			UCB < 17 µmol/l			UCB ≥ 17.1 µmol/l			*p*-Value
	**n**	**Mean (SD)**		**n**	**Mean (SD)**		**n**	**Mean (SD)**		
UCB (µmol/l)	2489	**6.393**	(3.61)	2442	**6.107**	(2.93)	47	**21.24**	(4.30)	***<0.001***
Age (years)	2489	**58.49**	(10.6)	2442	**58.54**	(10.6)	47	**55.97**	(11.4)	0.162
BMI (kg/m²)	2489	**26.31**	(4.43)	2442	**26.35**	(4.44)	47	**24.31**	(3.23)	***0.002***
Weight (kg)	2489	**75.15**	(14.8)	2442	**75.16**	(14.8)	47	**74.66**	(16.7)	0.942
Height (cm)	2489	**168.8**	(9.41)	2442	**168.7**	(9.34)	47	**174.2**	(11.8)	***<0.001***
WC (cm)	2489	**92.62**	(12.5)	2442	**92.69**	(12.5)	47	**89.04**	(12.9)	0.103
WHR	2489	**0.907**	(0.08)	2442	**0.907**	(0.08)	47	**0.907**	(0.08)	0.861
Heart rate (bpm)	2489	**70.18**	(11.1)	2442	**70.22**	(11.1)	47	**67.96**	(12.0)	***0.049***
BP diastolic (mmHg)	2489	**81.00**	(11.0)	2442	**80.99**	(11.0)	47	**81.36**	(11.5)	0.754
BP systolic (mmHg)	2489	**136.0**	(20.1)	2442	**136.0**	(20.1)	47	**134.1**	(19.9)	0.412
Left hand power (kg)	2478	**32.79**	(10.8)	2432	**32.70**	(10.8)	46	**37.63**	(11.1)	***0.003***
Right hand power (kg)	2485	**34.30**	(11.1)	2438	**34.22**	(11.1)	47	**38.81**	(12.1)	***0.005***
HbA1c (%)	2470	**6.043**	(0.59)	2423	**6.047**	(0.60)	47	**5.866**	(0.40)	***0.028***
Insulin (µU/mL)	2487	**6.128**	(4.77)	2440	**6.159**	(4.80)	47	**4.553**	(2.20)	***0.008***
Triglycerides (mmol/l)	2326	**1.271**	(0.87)	2283	**1.276**	(0.87)	43	**1.028**	(0.50)	***0.026***
Cholesterol (mmol/l)	2327	**5.609**	(1.03)	2284	**5.617**	(1.03)	43	**5.224**	(0.90)	***0.017***
HDL Cholesterol (mmol/l)	2360	**1.537**	(0.44)	2317	**1.537**	(0.44)	43	**1.532**	(0.40)	0.924
LDL Cholesterol (mmol/l)	2361	**3.331**	(0.87)	2318	**3.336**	(0.87)	43	**3.046**	(0.82)	***0.036***
Adiponectin (ng/ml)	2487	**14.35**	(7.65)	2440	**14.35**	(7.66)	47	**14.64**	(7.44)	0.749
FRS (points)	2246	**12.98**	(4.17)	2208	**13.02**	(4.16)	38	**10.68**	(4.08)	***0.002***
ALT (U/l)	2487	**24.18**	(10.3)	2440	**24.20**	(10.3)	47	**23.22**	(7.36)	0.882
γ-GT (U/l)	2346	**22.36**	(28.0)	2304	**22.33**	(27.6)	42	**24.28**	(43.8)	0.925
Ascorbic acid (mg/l)	2489	**5.177**	(3.65)	2442	**5.198**	(3.67)	47	**4.063**	(2.75)	***0.026***
Uric acid (mg/l)	2489	**45.45**	(12.0)	2442	**45.44**	(12.0)	47	**45.92**	(12.6)	0.839
Glutathione (µmol/l)	2488	**1107**	(196)	2441	**1106**	(197)	47	**1160**	(168)	***0.039***
Cysteine (µmol/l)	2488	**143.9**	(33.4)	2441	**144.1**	(33.4)	47	**131.0**	(28.1)	***0.008***
MDA (µmol/l)	2489	**0.322**	(0.23)	2442	**0.322**	(0.23)	47	**0.311**	(0.17)	0.891
Protein carbonyls (nmol/mg)	2489	**0.582**	(0.10)	2442	**0.581**	(0.10)	47	**0.601**	(0.10)	0.25
3-Nitrotyrosine (pmol/mg)	2470	**4.491**	(2.80)	2423	**4.494**	(2.80)	47	**4.313**	(2.36)	0.953
Urinary 8-isoprostane (ng/ml)	2480	**2.316**	(2.32)	2434	**2.322**	(2.34)	46	**1.984**	(0.87)	0.343
Plasma creatinine (µmol/l)	2487	**74.04**	(15.9)	2440	**73.96**	(15.8)	47	**78.49**	(17.8)	***0.039***
Urinary creatinine (pmol/l)	2480	**10.35**	(5.83)	2434	**10.32**	(5.79)	46	**11.90**	(7.51)	0.229
CRP (mg/l)	2487	**2.199**	(3.27)	2440	**2.210**	(3.29)	47	**1.590**	(2.12)	0.055
Arg-Pyr (%)	2441	**0.313**	(0.78)	2394	**0.312**	(0.78)	47	**0.356**	(0.74)	0.334
CML (%)	2441	**0.275**	(0.80)	2394	**0.274**	(0.79)	47	**0.329**	(1.35)	0.126

Data are presented as mean ± standard deviation. **Abbreviations**: UCB: unconjugated Bilirubin; RASIG: randomly recruited age-stratified individuals from the general population; GO: GEHA (genetics of healthy ageine) offspring; SGO: Spouses of GO (GEHA offspring); FRS: Framingham Risk Score; ALT: Alaninaminotransferase; γ-GT: Gamma glutamyl transferase; MDA: Malondialdehyde; CRP: C-reactive protein; Arg-Pyr: Arg-Pyrimidin; CML: Carboxymethyllysine.

Table S2: *Biomarkers of the study population separated by sex, related to Table 3*

Parameters	Total			Females			Males			*p*-Value
	n	Mean (SD)		n	Mean (SD)		n	Mean (SD)		
UCB (µmol/l)	2489	**6.393**	(3.61)	1361	**5.814**	(3.20)	1128	**7.092**	(3.93)	***<0.001***
Age (years)	2489	**58.49**	(10.6)	1361	**58.29**	(10.5)	1128	**58.73**	(10.7)	0.248
BMI (kg/m²)	2489	**26.31**	(4.43)	1361	**25.83**	(4.86)	1128	**26.90**	(3.77)	***<0.001***
Weight (kg)	2489	**75.15**	(14.8)	1361	**68.48**	(13.1)	1128	**83.21**	(12.6)	***<0.001***
Height (cm)	2489	**168.8**	(9.41)	1361	**162.9**	(6.57)	1128	**175.9**	(7.18)	***<0.001***
WC (cm)	2489	**92.62**	(12.5)	1361	**88.76**	(12.8)	1128	**97.28**	(10.4)	***<0.001***
WHR	2489	**0.907**	(0.08)	1361	**0.872**	(0.07)	1128	**0.948**	(0.06)	***<0.001***
Heart rate (bpm)	2489	**70.18**	(11.1)	1361	**71.31**	(10.2)	1128	**68.81**	(11.9)	***<0.001***
BP diastolic (mmHg)	2489	**81.00**	(11.0)	1361	**78.92**	(10.6)	1128	**83.51**	(10.9)	***<0.001***
BP systolic (mmHg)	2489	**136.0**	(20.1)	1361	**132.3**	(20.0)	1128	**140.4**	(19.3)	***<0.001***
Left hand power (kg)	2478	**32.79**	(10.8)	1355	**25.55**	(5.70)	1123	**41.52**	(8.99)	***<0.001***
Right hand power (kg)	2485	**34.30**	(11.1)	1359	**27.14**	(6.19)	1126	**42.95**	(9.42)	***<0.001***
HbA1c (%)	2470	**6.043**	(0.59)	1352	**6.073**	(0.57)	1118	**6.008**	(0.61)	***<0.001***
Insulin (µU/mL)	2487	**6.128**	(4.77)	1361	**5.729**	(4.46)	1126	**6.611**	(5.07)	***<0.001***
Triglycerides (mmol/l)	2326	**1.271**	(0.87)	1297	**1.150**	(0.73)	1029	**1.423**	(0.99)	***<0.001***
Cholesterol (mmol/l)	2327	**5.609**	(1.03)	1298	**5.710**	(1.02)	1029	**5.483**	(1.04)	***<0.001***
HDL Cholesterol (mmol/l)	2360	**1.537**	(0.44)	1316	**1.689**	(0.44)	1044	**1.344**	(0.36)	***<0.001***
LDL Cholesterol (mmol/l)	2361	**3.331**	(0.87)	1317	**3.309**	(0.87)	1044	**3.359**	(0.87)	0.074
Adiponectin (ng/ml)	2487	**14.35**	(7.65)	1360	**17.21**	(8.01)	1127	**10.90**	(5.48)	***<0.001***
FRS (points)	2246	**12.98**	(4.17)	1249	**13.96**	(4.54)	997	**11.75**	(3.26)	***<0.001***
ALT (U/l)	2487	**24.18**	(10.3)	1361	**21.72**	(8.44)	1126	**27.16**	(11.4)	***<0.001***
γ-GT (U/l)	2346	**22.36**	(28.0)	1304	**17.25**	(24.4)	1042	**28.76**	(30.7)	***<0.001***
Ascorbic acid (mg/l)	2489	**5.177**	(3.65)	1361	**5.756**	(3.74)	1128	**4.477**	(3.42)	***<0.001***
Uric acid (mg/l)	2489	**45.45**	(12.0)	1361	**40.52**	(10.0)	1128	**51.39**	(11.5)	***<0.001***
Glutathione (µmol/l)	2488	**1107**	(196)	1360	**1099**	(191)	1128	**1116**	(202)	***0.037***
Cysteine (µmol/l)	2488	**143.9**	(33.4)	1360	**147.2**	(33.1)	1128	**139.9**	(33.3)	***<0.001***
MDA (µmol/l)	2489	**0.322**	(0.23)	1361	**0.307**	(0.21)	1128	**0.339**	(0.25)	***<0.001***
Protein carbonyls (nmol/mg)	2489	**0.582**	(0.10)	1361	**0.581**	(0.09)	1128	**0.582**	(0.11)	0.819
3-Nitrotyrosine (pmol/mg)	2470	**4.491**	(2.80)	1351	**4.538**	(2.84)	1119	**4.434**	(2.75)	0.424
Urinary 8-isoprostane (ng/ml)	2480	**2.316**	(2.32)	1356	**2.604**	(2.41)	1124	**1.969**	(2.15)	***<0.001***
Plasma creatinine (µmol/l)	2487	**74.04**	(15.9)	1361	**66.58**	(12.2)	1126	**83.06**	(15.1)	***<0.001***
Urinary creatinine (pmol/l)	2480	**10.35**	(5.83)	1356	**8.751**	(5.26)	1124	**12.28**	(5.89)	***<0.001***
CRP (mg/l)	2487	**2.199**	(3.27)	1361	**2.214**	(3.22)	1126	**2.180**	(3.33)	0.496
Arg-Pyr (%)	2441	**0.313**	(0.78)	1331	**0.315**	(0.87)	1110	**0.312**	(0.65)	0.624
CML (%)	2441	**0.275**	(0.80)	1331	**0.264**	(0.73)	1110	**0.288**	(0.88)	0.502

Data are presented as mean ± standard deviation. **Abbreviations**: UCB: unconjugated Bilirubin; RASIG: randomly recruited age-stratified individuals from the general population; GO: GEHA (genetics of healthy ageine) offspring; SGO: Spouses of GO (GEHA offspring); FRS: Framingham Risk Score; ALT: Alaninaminotransferase; γ-GT: Gamma glutamyl transferase; MDA: Malondialdehyde; CRP: C-reactive protein; Arg-Pyr: Arg-Pyrimidin; CML: Carboxymethyllysine.

Table S3: *Biomarkers of the UCB subgroups separated by sex, related to Table 3*

**Parameter**	**Class 1 (N = 248)**							**Class 10 (N = 248)**						
	Females (0)			Males (1)			***p*-Value**	Females (0)			Males (1)			***p*-Value**
	**n**	**Mean (SD)**		**n**	**Mean (SD)**			**n**	**Mean (SD)**		**n**	**Mean (SD)**		
UCB (µmol/l)	156	**2.499**	(0.39)	92	**2.434**	(0.49)	0.627	91	**14.46**	(4.23)	157	**14.71**	(3.91)	0.289
Age (years)	156	**58.31**	(10.6)	92	**57.94**	(10.4)	0.629	91	**56.54**	(12.2)	157	**59.09**	(11.1)	0.121
BMI (kg/m²)	156	**26.30**	(5.38)	92	**28.50**	(4.48)	***<0.001***	91	**24.40**	(4.58)	157	**26.40**	(3.79)	***<0.001***
Weight (kg)	156	**68.57**	(14.0)	92	**86.39**	(13.6)	***<0.001***	91	**65.35**	(13.2)	157	**82.34**	(12.6)	***<0.001***
Height (cm)	156	**161.6**	(6.12)	92	**174.2**	(6.21)	***<0.001***	91	**163.5**	(6.73)	157	**176.6**	(7.70)	***<0.001***
WC (cm)	156	**90.21**	(13.7)	92	**100.7**	(10.7)	***<0.001***	91	**86.53**	(12.8)	157	**96.17**	(10.3)	***<0.001***
WHR	156	**0.881**	(0.07)	92	**0.954**	(0.05)	***<0.001***	91	**0.864**	(0.07)	157	**0.948**	(0.06)	***<0.001***
Heart rate (bpm)	156	**72.97**	(9.99)	92	**73.20**	(12.5)	0.965	91	**70.88**	(9.56)	157	**67.19**	(12.0)	***0.002***
BP diastolic (mmHg)	156	**80.69**	(10.0)	92	**83.29**	(11.8)	0.145	91	**77.24**	(9.60)	157	**83.59**	(10.2)	***<0.001***
BP systolic (mmHg)	156	**134.5**	(21.1)	92	**140.4**	(20.5)	***0.031***	91	**129.0**	(19.1)	157	**140.9**	(19.9)	***<0.001***
Left hand power (kg)	154	**24.86**	(5.11)	90	**42.51**	(8.76)	***<0.001***	90	**26.73**	(7.18)	157	**41.36**	(8.55)	***<0.001***
Right hand power (kg)	156	**26.40**	(5.51)	92	**43.12**	(9.54)	***<0.001***	91	**28.49**	(7.77)	157	**42.62**	(8.76)	***<0.001***
HbA1c (%)	154	**6.142**	(0.80)	89	**6.135**	(0.59)	0.687	90	**5.984**	(0.42)	155	**5.880**	(0.51)	***0.048***
Insulin (µU/mL)	156	**6.288**	(5.01)	91	**9.418**	(7.06)	***<0.001***	91	**4.516**	(2.22)	157	**5.332**	(3.11)	0.063
Triglycerides (mmol/l)	147	**1.211**	(1.04)	86	**1.941**	(1.79)	***<0.001***	84	**0.950**	(0.39)	144	**1.203**	(0.63)	***0.002***
Cholesterol (mmol/l)	147	**5.677**	(0.97)	86	**5.472**	(0.94)	***0.059***	84	**5.577**	(1.02)	144	**5.259**	(1.05)	***0.028***
HDL Cholesterol (mmol/l)	149	**1.595**	(0.45)	88	**1.181**	(0.33)	***<.001***	86	**1.785**	(0.45)	145	**1.375**	(0.35)	***<0.001***
LDL Cholesterol (mmol/l)	149	**3.363**	(0.85)	88	**3.444**	(0.81)	0.424	86	**3.140**	(0.90)	145	**3.170**	(0.88)	0.476
Adiponectin (ng/ml)	156	**17.33**	(9.39)	92	**9.811**	(4.44)	***<0.001***	91	**19.24**	(8.65)	156	**11.90**	(6.18)	***<0.001***
FRS (points)	140	**14.74**	(3.78)	80	**12.46**	(2.84)	***<0.001***	77	**12.71**	(5.50)	134	**11.13**	(3.81)	***0.005***
ALT (U/l)	156	**21.70**	(9.30)	91	**27.10**	(11.3)	***<0.001***	91	**20.45**	(5.94)	157	**25.46**	(10.1)	***<0.001***
γ-GT (U/l)	148	**20.57**	(36.8)	88	**30.87**	(25.9)	***<0.001***	83	**16.33**	(18.6)	145	**24.75**	(30.4)	***<0.001***
Ascorbic acid (mg/l)	156	**5.848**	(3.56)	92	**5.208**	(6.99)	***0.002***	91	**5.235**	(3.06)	157	**4.367**	(2.77)	***0.03***
Uric acid (mg/l)	156	**38.87**	(10.2)	92	**51.81**	(10.7)	***<0.001***	91	**39.22**	(10.8)	157	**51.11**	(11.4)	***<0.001***
Glutathione (µmol/l)	156	**1085**	(168)	92	**1101**	(229)	0.461	91	**1119**	(200)	157	**1142**	(203)	0.345
Cysteine (µmol/l)	156	**145.9**	(35.9)	92	**139.5**	(42.5)	0.078	91	**143.2**	(29.8)	157	**135.5**	(31.3)	0.094
MDA (µmol/l)	156	**0.295**	(0.20)	92	**0.340**	(0.25)	0.326	91	**0.319**	(0.21)	157	**0.324**	(0.19)	0.756
Protein carbonyls (nmol/mg)	156	**0.581**	(0.09)	92	**0.605**	(0.25)	0.868	91	**0.589**	(0.08)	157	**0.587**	(0.08)	0.924
3-Nitrotyrosine (pmol/mg)	153	**4.699**	(3.34)	89	**4.368**	(2.41)	0.911	90	**4.604**	(2.32)	156	**4.252**	(2.42)	0.156
Urinary 8-isoprostane (ng/ml)	155	**3.119**	(3.04)	91	**2.512**	(4.25)	***<0.001***	91	**2.578**	(1.84)	156	**1.749**	(0.74)	***<0.001***
Plasma creatinine (µmol/l)	156	**63.17**	(11.7)	91	**82.91**	(22.1)	***<0.001***	91	**67.28**	(12.7)	157	**84.94**	(13.0)	***<0.001***
Urinary creatinine (pmol/l)	155	**8.043**	(4.39)	91	**12.37**	(6.26)	***<0.001***	91	**9.143**	(5.53)	156	**12.50**	(6.05)	***<0.001***
CRP (mg/l)	156	**2.245**	(2.60)	91	**3.383**	(4.00)	***0.017***	91	**2.046**	(3.73)	157	**2.102**	(4.22)	0.384
Arg-Pyr (%)	150	**0.252**	(0.42)	92	**0.272**	(0.21)	***0.018***	89	**0.554**	(1.98)	153	**0.317**	(0.58)	0.808
CML (%)	150	**0.209**	(0.53)	92	**0.162**	(0.15)	0.764	89	**0.166**	(0.40)	153	**0.303**	(1.05)	0.187

Data are presented as mean ± standard deviation. **Abbreviations**: UCB: unconjugated Bilirubin; RASIG: randomly recruited age-stratified individuals from the general population; GO: GEHA (genetics of healthy ageine) offspring; SGO: Spouses of GO (GEHA offspring); FRS: Framingham Risk Score; ALT: Alaninaminotransferase; γ-GT: Gamma glutamyl transferase; MDA: Malondialdehyde; CRP: C-reactive protein; Arg-Pyr: Arg-Pyrimidin; CML: Carboxymethyllysine.

Table S4: *Biomarkers of the study population separated by age (cut-off 50 years), related to Table 4*

Parameters	Total			Age < 50 years			Age ≥ 50 years			*p*-Value
	n	Mean (SD)		n	Mean (SD)		n	Mean (SD)		
UCB (µmol/l)	2489	**6.393**	(3.61)	569	**6.776**	(3.98)	1920	**6.280**	(3.48)	***0.015***
Age (years)	2489	**58.49**	(10.6)	569	**42.75**	(4.48)	1920	**63.15**	(6.66)	***<0.001***
BMI (kg/m²)	2489	**26.31**	(4.43)	569	**24.99**	(3.94)	1920	**26.71**	(4.49)	***<0.001***
Weight (kg)	2489	**75.15**	(14.8)	569	**73.73**	(15.0)	1920	**75.57**	(14.7)	***0.011***
Height (cm)	2489	**168.8**	(9.41)	569	**171.3**	(9.81)	1920	**168.0**	(9.16)	***<0.001***
WC (cm)	2489	**92.62**	(12.5)	569	**87.96**	(12.0)	1920	**94.00**	(12.4)	***<0.001***
WHR	2489	**0.907**	(0.08)	569	**0.882**	(0.08)	1920	**0.914**	(0.08)	***<0.001***
Heart rate (bpm)	2489	**70.18**	(11.1)	569	**70.51**	(11.4)	1920	**70.08**	(11.0)	0.258
BP diastolic (mmHg)	2489	**81.00**	(11.0)	569	**78.08**	(10.9)	1920	**81.86**	(10.9)	***<0.001***
BP systolic (mmHg)	2489	**136.0**	(20.1)	569	**125.8**	(16.4)	1920	**139.0**	(20.0)	***<0.001***
Left hand power (kg)	2478	**32.79**	(10.8)	569	**36.01**	(10.8)	1909	**31.83**	(10.7)	***<0.001***
Right hand power (kg)	2485	**34.30**	(11.1)	569	**37.23**	(11.1)	1916	**33.43**	(11.0)	***<0.001***
HbA1c (%)	2470	**6.043**	(0.59)	566	**5.884**	(0.44)	1904	**6.091**	(0.62)	***<0.001***
Insulin (µU/mL)	2487	**6.128**	(4.77)	569	**5.597**	(4.51)	1918	**6.286**	(4.83)	***<0.001***
Triglycerides (mmol/l)	2326	**1.271**	(0.87)	527	**1.188**	(0.88)	1799	**1.295**	(0.86)	***<0.001***
Cholesterol (mmol/l)	2327	**5.609**	(1.03)	527	**5.380**	(0.93)	1800	**5.677**	(1.05)	***<0.001***
HDL Cholesterol (mmol/l)	2360	**1.537**	(0.44)	542	**1.497**	(0.42)	1818	**1.548**	(0.44)	***0.023***
LDL Cholesterol (mmol/l)	2361	**3.331**	(0.87)	542	**3.179**	(0.82)	1819	**3.376**	(0.88)	***<0.001***
Adiponectin (ng/ml)	2487	**14.35**	(7.65)	569	**12.88**	(6.36)	1918	**14.79**	(7.94)	***<0.001***
FRS (points)	2246	**12.98**	(4.17)	511	**8.249**	(4.37)	1735	**14.37**	(2.89)	***<0.001***
ALT (U/l)	2487	**24.18**	(10.3)	569	**24.03**	(11.2)	1918	**24.22**	(9.97)	***0.011***
γ-GT (U/l)	2346	**22.36**	(28.0)	533	**19.80**	(29.3)	1813	**23.11**	(27.5)	***<0.001***
Ascorbic acid (mg/l)	2489	**5.177**	(3.65)	569	**4.848**	(3.17)	1920	**5.274**	(3.78)	***0.028***
Uric acid (mg/l)	2489	**45.45**	(12.0)	569	**43.13**	(11.7)	1920	**46.14**	(12.0)	***<0.001***
Glutathione (µmol/l)	2488	**1107**	(196)	568	**1105**	(192)	1920	**1107**	(197)	0.998
Cysteine (µmol/l)	2488	**143.9**	(33.4)	568	**132.1**	(29.9)	1920	**147.4**	(33.5)	***<0.001***
MDA (µmol/l)	2489	**0.322**	(0.23)	569	**0.299**	(0.20)	1920	**0.328**	(0.24)	***0.026***
Protein carbonyls (nmol/mg)	2489	**0.582**	(0.10)	569	**0.587**	(0.13)	1920	**0.580**	(0.09)	0.538
3-Nitrotyrosine (pmol/mg)	2470	**4.491**	(2.80)	568	**4.586**	(2.99)	1902	**4.462**	(2.74)	0.691
Urinary 8-isoprostane (ng/ml)	2480	**2.316**	(2.32)	568	**2.017**	(1.57)	1912	**2.405**	(2.49)	***<0.001***
Plasma creatinine (µmol/l)	2487	**74.04**	(15.9)	569	**73.05**	(14.3)	1918	**74.34**	(16.3)	0.27
Urinary creatinine (pmol/l)	2480	**10.35**	(5.83)	568	**11.44**	(6.73)	1912	**10.03**	(5.49)	***<0.001***
CRP (mg/l)	2487	**2.199**	(3.27)	569	**1.809**	(2.86)	1918	**2.314**	(3.37)	***<0.001***
Arg-Pyr (%)	2441	**0.313**	(0.78)	564	**0.314**	(0.90)	1877	**0.313**	(0.74)	0.312
CML (%)	2441	**0.275**	(0.80)	564	**0.227**	(0.60)	1877	**0.289**	(0.85)	***0.011***

Data are presented as mean ± standard deviation. **Abbreviations**: UCB: unconjugated Bilirubin; RASIG: randomly recruited age-stratified individuals from the general population; GO: GEHA (genetics of healthy ageine) offspring; SGO: Spouses of GO (GEHA offspring); FRS: Framingham Risk Score; ALT: Alaninaminotransferase; γ-GT: Gamma glutamyl transferase; MDA: Malondialdehyde; CRP: C-reactive protein; Arg-Pyr: Arg-Pyrimidin; CML: Carboxymethyllysine.

Table S5: *Biomarkers of the UCB subgroups separated by age (cut-off 50 years), related to Table 4*

**Parameter**	**Class 1 (N = 248)**							**Class 10 (N = 248)**						
	Age < 50 years			Age ≥ 50 years			***p*-Value**	Age < 50 years			Age ≥ 50 years			***p*-Value**
	**N**	**Mean (SD)**		**N**	**Mean (SD)**			**N**	**Mean (SD)**		**N**	**Mean (SD)**		
UCB (µmol/l)	58	**2.434**	(0.48)	190	**2.488**	(0.41)	0.774	72	**14.91**	(4.17)	176	**14.50**	(3.96)	0.549
Age (years)	58	**43.06**	(4.54)	190	**62.79**	(6.77)	***<0.001***	72	**42.70**	(4.20)	176	**64.48**	(6.57)	***<0.001***
BMI (kg/m²)	58	**25.95**	(4.42)	190	**27.47**	(5.33)	0.066	72	**24.11**	(3.71)	176	**26.30**	(4.23)	***<0.001***
Weight (kg)	58	**73.17**	(16.9)	190	**75.79**	(16.1)	0.239	72	**73.32**	(15.8)	176	**77.24**	(14.9)	***0.045***
Height (cm)	58	**167.2**	(9.15)	190	**166.0**	(8.50)	0.602	72	**173.7**	(10.2)	176	**171.1**	(9.40)	0.087
WC (cm)	58	**89.53**	(13.1)	190	**95.47**	(13.5)	***0.003***	72	**87.18**	(11.6)	176	**94.86**	(11.7)	***<0.001***
WHR	58	**0.886**	(0.08)	190	**0.915**	(0.07)	***0.006***	72	**0.885**	(0.07)	176	**0.931**	(0.07)	***<0.001***
Heart rate (bpm)	58	**75.57**	(10.6)	190	**72.28**	(11.0)	***0.035***	72	**67.75**	(12.2)	176	**68.87**	(10.9)	0.366
BP diastolic (mmHg)	58	**80.59**	(11.5)	190	**81.98**	(10.5)	0.242	72	**76.29**	(8.77)	176	**83.30**	(10.3)	***<0.001***
BP systolic (mmHg)	58	**126.3**	(16.6)	190	**139.8**	(21.3)	***<0.001***	72	**124.3**	(13.9)	176	**141.5**	(20.6)	***<0.001***
Left hand power (kg)	58	**34.84**	(10.5)	186	**30.28**	(10.7)	***0.002***	72	**38.50**	(10.2)	175	**35.01**	(10.8)	***0.025***
Right hand power (kg)	58	**36.12**	(10.6)	190	**31.53**	(10.7)	***<0.001***	72	**40.01**	(11.1)	176	**36.39**	(10.6)	***0.049***
HbA1c (%)	58	**5.979**	(0.45)	185	**6.189**	(0.80)	0.073	72	**5.848**	(0.41)	173	**5.948**	(0.50)	0.218
Insulin (µU/mL)	58	**7.093**	(5.67)	189	**7.548**	(6.15)	0.618	72	**4.696**	(2.65)	176	**5.171**	(2.91)	0.22
Triglycerides (mmol/l)	55	**1.582**	(1.67)	178	**1.449**	(1.32)	0.735	67	**0.988**	(0.60)	161	**1.160**	(0.54)	***<0.001***
Cholesterol (mmol/l)	55	**5.492**	(1.06)	178	**5.635**	(0.93)	0.333	67	**5.216**	(0.82)	161	**5.443**	(1.13)	0.145
HDL Cholesterol (mmol/l)	57	**1.373**	(0.44)	180	**1.463**	(0.46)	0.133	68	**1.507**	(0.34)	163	**1.536**	(0.47)	0.978
LDL Cholesterol (mmol/l)	57	**3.344**	(0.90)	180	**3.409**	(0.82)	0.395	68	**3.061**	(0.77)	163	**3.199**	(0.93)	0.229
Adiponectin (ng/ml)	58	**12.94**	(8.52)	190	**15.03**	(8.73)	***0.02***	72	**13.16**	(5.86)	175	**15.20**	(8.68)	0.18
FRS (points)	53	**10.70**	(3.97)	167	**14.93**	(2.85)	***<0.001***	61	**6.660**	(4.08)	150	**13.77**	(2.79)	***<0.001***
ALT (U/l)	58	**23.12**	(10.9)	189	**23.87**	(10.3)	0.455	72	**23.06**	(9.11)	176	**23.85**	(9.14)	0.314
γ-GT (U/l)	56	**25.72**	(38.2)	180	**24.00**	(31.9)	0.786	65	**15.73**	(14.9)	163	**24.06**	(30.2)	***0.006***
Ascorbic acid (mg/l)	58	**5.354**	(3.95)	190	**5.689**	(5.41)	0.757	72	**4.692**	(2.86)	176	**4.682**	(2.94)	0.891
Uric acid (mg/l)	58	**42.27**	(13.6)	190	**44.10**	(11.6)	0.234	72	**44.13**	(13.6)	176	**47.82**	(12.0)	***0.012***
Glutathione (µmol/l)	58	**1065**	(225)	190	**1099**	(181)	0.294	72	**1104**	(176)	176	**1146**	(210)	0.147
Cysteine (µmol/l)	58	**123.1**	(33.7)	190	**149.7**	(37.8)	***<0.001***	72	**126.7**	(27.1)	176	**143.0**	(31.1)	***<0.001***
MDA (µmol/l)	58	**0.292**	(0.20)	190	**0.318**	(0.23)	0.526	72	**0.287**	(0.19)	176	**0.336**	(0.20)	***0.047***
Protein carbonyls (nmol/mg)	58	**0.596**	(0.30)	190	**0.588**	(0.10)	0.066	72	**0.595**	(0.09)	176	**0.585**	(0.08)	0.485
3-Nitrotyrosine (pmol/mg)	57	**4.493**	(2.45)	185	**4.603**	(3.19)	0.567	72	**4.532**	(2.67)	174	**4.319**	(2.26)	0.52
Urinary 8-isoprostane (ng/ml)	58	**2.064**	(0.85)	188	**3.151**	(3.98)	0.064	72	**2.042**	(1.02)	175	**2.060**	(1.43)	0.933
Plasma creatinine (µmol/l)	58	**66.67**	(13.8)	189	**71.60**	(20.0)	0.151	72	**78.39**	(14.2)	176	**78.49**	(16.0)	0.792
Urinary creatinine (pmol/l)	58	**10.43**	(5.94)	188	**9.402**	(5.43)	0.261	72	**11.86**	(6.50)	175	**11.02**	(5.89)	0.305
CRP (mg/l)	58	**1.996**	(2.27)	189	**2.870**	(3.45)	0.158	72	**1.827**	(4.36)	176	**2.186**	(3.91)	***<0.001***
Arg-Pyr (%)	58	**0.280**	(0.62)	184	**0.253**	(0.21)	0.201	71	**0.288**	(0.39)	171	**0.452**	(1.51)	0.319
CML (%)	58	**0.155**	(0.14)	184	**0.203**	(0.49)	0.557	71	**0.188**	(0.46)	171	**0.280**	(0.99)	***0.021***

Data are presented as mean ± standard deviation. **Abbreviations**: UCB: unconjugated Bilirubin; RASIG: randomly recruited age-stratified individuals from the general population; GO: GEHA (genetics of healthy ageine) offspring; SGO: Spouses of GO (GEHA offspring); FRS: Framingham Risk Score; ALT: Alaninaminotransferase; γ-GT: Gamma glutamyl transferase; MDA: Malondialdehyde; CRP: C-reactive protein; Arg-Pyr: Arg-Pyrimidin; CML: Carboxymethyllysine.
